# Clinicopathologic Features and Risk Factors of Proteinuria in Transplant Glomerulopathy

**DOI:** 10.3389/fmed.2021.666319

**Published:** 2021-07-02

**Authors:** Qiang Zhang, Klemens Budde, Danilo Schmidt, Fabian Halleck, Michael Duerr, Marcel G. Naik, Manuel Mayrdorfer, Wiebke Duettmann, Frederick Klauschen, Birgit Rudolph, Kaiyin Wu

**Affiliations:** ^1^Department of Nephrology and Medical Intensive Care, Charité-Universitätsmedizin Berlin, Berlin, Germany; ^2^Department of Organ Transplant, The First Affiliated Hospital, Sun Yat-sen University, Guangzhou, China; ^3^Department of Pathology, Charité-Universitätsmedizin Berlin, Berlin, Germany

**Keywords:** blood pressure, graft function, kidney transplantation, proteinuria, renal biopsy

## Abstract

**Background:** Transplant glomerulopathy (TG) is one of the main causes of post-transplant proteinuria (PU). The features and possible risk factors for proteinuria in TG patients are uncertain.

**Methods:** We investigated all patients who had biopsy-proven TG from 2000 to 2018 in our center. The clinical and histological data were compared between two groups with or without PU (cut-off = 0.3 g/day). Spearman correlation analysis was used to evaluate the relationship between PU and pathological changes. The risk factors for PU in TG patients were determined by multivariable logistic regression analysis.

**Results:** One hundred and twenty-five (75.76%) of all enrolled 165 TG patients had proteinuria ≥0.3 g/day at the time of biopsy. TG patients' PU level was significantly correlated with Banff lesion score cg (ρ = 0.247, *P* = 0.003), and mm (ρ = 0.257, *P* = 0.012). Systolic blood pressure ≥140 mmHg (OR 2.72, 95% CI 1.04–7.10, *P* = 0.041), diastolic blood pressure ≥90 mmHg (OR 4.84, 95% CI 1.39–16.82, *P* = 0.013), peak PRA ≥5% (OR 6.47, 95% CI 1.67–25.01, *P* = 0.007), positive C4d staining (OR 4.55, 95% CI 1.29–16.11, 0.019), tacrolimus-based regimen (OR 3.5, 95% CI 1.28–9.54, *P* = 0.014), and calcium channel blocker usage (OR 4.38, 95% CI 1.59–12.09, *P* = 0.004) were independent risk factors for PU.

**Conclusions:** Proteinuria is common in TG patients. systolic blood pressure ≥140 mmHg, diastolic blood pressure ≥90 mmHg, peak PRA ≥5%, positive C4d staining, tacrolimus-based regimen, and calcium channel blocker usage are associated with proteinuria in TG patients.

## Introduction

Transplant glomerulopathy (TG) is characterized by glomerular basement membrane (GBM) double contours (Banff chronic allograft glomerulopathy score >0) as a result of chronic endothelial cell injury by various mechanisms (1–3). The incidence of TG increases over time after kidney transplantation, and a protocol biopsy-based study demonstrated that TG was present in up to 20% of kidney transplant recipients at 5 years following transplantation (4). TG is an independent risk factor for graft loss; meanwhile, other factors (serum creatinine, proteinuria, C4d positivity) can also affect the prognosis of TG patients (5–7). Graft survival of TG patients with a higher level of proteinuria was much worse compared to those with less proteinuria (6, 8).

Proteinuria is common after kidney transplantation and is also one of the most cardinal features of TG (9). Moreover, the degree of proteinuria is the main risk factor for graft loss regardless of allograft function or underlying pathological change in kidney transplant recipients (10, 11). Meanwhile, the current treatment for TG has limited effect on improving long-term allograft survival, management of proteinuria in TG patients might offer a favorable prognosis (12). Therefore, it is of great importance to identify the factors that can influence the production of proteinuria.

There is limited research specifically on proteinuria in patients with TG, and most of the existing studies focus on proteinuria as one of the prognostic risk factors for TG (5, 6, 13, 14). Both clinical and histologic risk factors that cause or exacerbate proteinuria in patients with TG remain to be explored in depth.

Thus, this study was designed to investigate the incidence, potential risk factors for proteinuria after the diagnosis of TG in kidney transplant recipients. Additionally, the clinicopathologic features of TG patients with proteinuria were assessed.

## Materials and Methods

### Study Design and Patient Population

A retrospective cross-sectional study was performed on kidney transplant recipients at Charité-Universitätsmedizin Berlin (Berlin, Germany) from 1st January 2000 to 31st December 2018. All study activities were following the Declaration of Helsinki 2000 and the Declaration of Istanbul 2018. All patients' information was obtained from the transplant database system (TBase) which is specially designed for kidney transplant patients under local regulation and European Union General Data Protection Regulation (15). The guidelines of the human research ethics committee of Charité—Universitätsmedizin Berlin were also obeyed throughout the whole process of this study.

All adult kidney transplant recipients with a biopsy-proven diagnosis of TG defined by the Banff consensus guideline were eligible for this study. Biopsies with secondary glomerular pathology that can cause glomerular basement membrane double contours were excluded, such as recurrent/*de novo* glomerulopathy, thrombotic microangiopathy (TMA), and hepatitis C virus-associated membranoproliferative glomerulonephritis (MPGN). Patients with mammalian target of rapamycin (mTOR) inhibitors-induced proteinuria were also excluded.

### Clinical and Laboratory Information

Data were extracted from the electronic transplant database and medical records in our center. TBase was established in 2,000 and clinical as well as laboratory data including urinary protein excretion were prospectively collected at each follow-up visit since then (16, 17). Urinary 24 h total protein remains the reference standard for proteinuria quantification in glomerulonephritis (18). Twenty-four-hour urine tests are available in 146 patients at the time of biopsy or 4 weeks around biopsy without special treatment. For patients (*n* = 17) without a 24-h urine test, spot urinary protein concentrations (mg/L) were converted to 24-h urine test unit (g/24 h) assuming 2L urinary output per day (19). In two patients, only dipstick's results were available at the time of biopsy. One result with negative dipstick was estimated to be 0.075 g/24 h (= the average urinary protein concentration between 0.0 and 0.15 g/24 h proteinuria) and the other with double-positive (++) dipsticks were imputed as 3.0 g/24 h (= the average urinary protein concentration between 1.5 and 4.5 g/24 h) (20).

### Biopsy and Histopathology

All biopsies in this study were performed for cause when clinically indicated (elevated serum creatinine and/or proteinuria). For recipients with multiple biopsies, only the first identified TG diagnosis was considered for enrollment in the study.

Formalin-fixed paraffin-embedded sections were cut at 3 μm and stained with HE, Masson's trichrome, periodic acid-Schiff, silver methenamine staining for light microscopy. C4d was detected on formalin-fixed tissue stained with polyclonal anti-C4d antibody (Dianova, Germany). Two pathologists (K.W. and B.R.) examined the biopsies independently, blinded to clinical data. All biopsy features were evaluated and scored semiquantitatively according to the Banff 2018 classification. These scores included grade of interstitial inflammation (i), tubulitis (t), intimal arteritis (v), glomerulitis (g), peritubular capillaritis (ptc), endothelial cells C4d staining extent of peritubular capillary and medullary *vasa recta* (C4d), interstitial fibrosis (ci), tubular atrophy (ct), vascular fibrous intimal thickening (cv), glomerular basement membrane double contours (cg), arteriolar hyalinosis (ah), and mesangial matrix expansion (mm).

### HLA Antibody Testing

All kidney transplantation was performed based on negative complement-dependent cytotoxicity crossmatch using T- and B-lymphocytes. Panel reactive antibody (PRA) was detected by the solid-phase assay. PRA was calculated as the percentage of positive reaction against a panel of HLA-antigens in donor blood.

All patients' serum samples were obtained at allograft dysfunction or once a year after transplantation since 2006 as previously described (21). Samples were qualitatively screened for anti-human leukocyte antigen (HLA) antibodies by Lambda Antigen Tray assay (One Lambda, Canoga Park, CA, USA) till 2005. Luminex-based bead assay LABScreen Mixed and LABScreen single antigen bead assay (One Lambda) were performed to screen anti-HLA antibodies (available from 2006). All the tests were performed according to the manufacturer's instructions. Anti-HLA antibodies against donor HLA were defined as donor-specific antibodies (DSAs). All normalized mean fluorescence intensity (MFI) units of more than 1,000 were considered positive. The immunodominant DSA results were assigned to the DSA with the highest MFI value at the time of diagnosis.

### Statistical Analysis

The normality of data was tested using the Kolmogorov-Smirnov test. Normally distributed data were expressed as mean ± standard deviations and were compared between two groups using Student's *t*-tests. Continuous variables from different distribution were described using medians (ranges) and were compared within two groups using the non-parametric Mann-Whitney *U*-test. Categorical variables were summarized by numbers (percentages) and were compared between two groups with Chi-squared test (or Fisher exact test if appropriate). The relationships between the level of proteinuria and Banff lesion scores were determined by Spearman correlation analysis. All tests were two-tailed.

The logistic regression model was developed and reported in accordance with recommendations for the assessment and reporting of multivariable logistic regression in transplantation literature (22). The individual effect of the variables on proteinuria was evaluated using a univariable logistic regression model by the “Enter” method. Significant predictors (*P* < 0.1) in the univariable analysis that were considered clinically relevant were fitted into a multivariable logistic regression model according to a forward selection, likelihood ratio test with an elimination threshold of 0.1. The Hosmer Lemeshow test was performed to test the goodness of fit for logistic regression models. The multicollinearity of covariables was tested by examining tolerance and the variance inflation factor. No collinearity was detected in our dataset after continuous variables were dichotomized. Internal validation was performed using 2,000 bootstrap samples to reduce overfit bias. The discriminative performance of models was evaluated using the area under the curve (AUC) of the receiver operator characteristic (ROC) analysis. An AUC value of 0.7–0.8 denotes a clinically useful test and a value of 0.8–0.9 is considered as an excellent predictive value. The performance of both the initial model and adjusted model was assessed in terms of discrimination and calibration (slope of the calibration line and intercept).

Statistical analyses were performed using IBM SPSS Statistics 26.0 (SPSS Inc., Chicago, IL), SPSS Modeler 12.0 (IBM, Armonk, NY, USA), and GraphPad Prism 8.0 (GraphPad Software, La Jolla, CA) software. *P* < 0.05 was considered to indicate statistically significant difference.

## Results

### Demographic and Clinical Characteristics of Enrolled Patients

**Figure 1:**
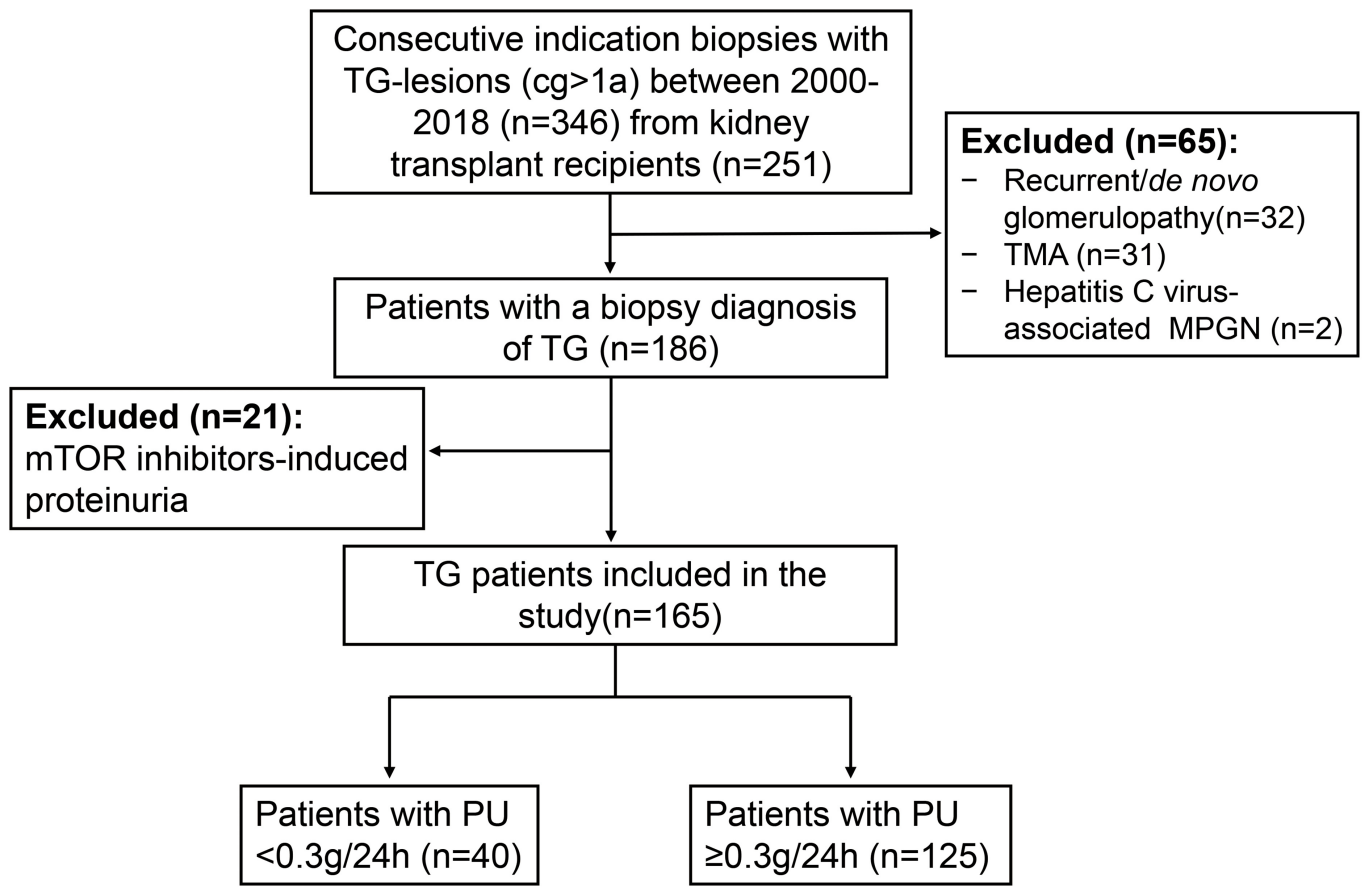
Flow chart of TG patients enrolled in the study. There were a total of 2,375 indication biopsies were performed due to elevated creatinine and/or proteinuria from 2000 to 2018 in our center. TG-lesions were found in 346 biopsies from 251 kidney transplant recipients. Biopsy cases with recurrent/*de novo* glomerulonephritis (*n* = 32), TMA (*n* = 31), hepatitis C-virus associated MPGN (*n* = 2) were excluded. After further excluding the patients with mTOR inhibitors-induced proteinuria (PU ≥ 0.3 g/day, *n* = 21), a total of 165 patients were enrolled in our study. TG, transplant glomerulopathy; cg, chronic allograft glomerulopathy; TMA, thrombotic microangiopathy; MPGN, membranoproliferative glomerulonephritis; mTOR, mammalian target of rapamycin; PU, proteinuria.

A total of 2,375 indication biopsies were performed due to elevated creatinine and/or proteinuria from Jan. 2000 to Dec. 2018 in our center. TG-lesions (cg >1a) were found in 346 biopsies (346/2375, 14.57%) from 251 kidney transplant recipients. Biopsy cases with recurrent/*de novo* glomerulonephritis (*n* = 32), TMA (*n* = 31), hepatitis C-virus associated MPGN (*n* = 2) were excluded. After further excluding the patients with mTOR inhibitors-induced proteinuria (PU ≥0.3 g/24 h, *n* = 21), a total of 165 patients were enrolled in this study (Figure 1).

The baseline demographics and clinical characteristics of the 165 patients with TG after kidney transplantation are presented in Table 1. TG patients underwent kidney transplantation with a mean age of 43.65 ± 15.18, which included 38 patients (38/165, 23.03%) who have at least one previous renal allograft. More than half of enrolled TG patients (94/165, 56.97%) are male and received deceased donor organs (101/165, 61.21%). The median cold ischemia time for deceased-donor transplantation was 11 h (from 3.5 to 31 h). Patients had a mean of 3.09 ± 1.42 HLA mismatches. The percentage of peak panel reactive antibody (PRA) in 35 patients (21.21%) and pre-transplant PRA in 19 patients (11.52%) were no <5%, respectively. The most common primary renal disease was glomerulonephritis (30/165, 18.18%), and the baseline serum creatinine after transplantation was 1.63 ± 0.64 mg/dL.

**Table 1:** Baseline demographics and clinical characteristics of TG patients.

**Variables**	**Overall (*n =* 165)**	**PU <0.3 g/24 h (*n =* 40)**	**PU ≥0.3 g/24 h (*n =* 125)**	*P*-value^*^
Age at the time of Tx (years)	43.65 ± 15.18	46.60 ± 15.37	42.71 ± 15.06	0.159
Male sex	94 (56.97)	21 (52.5)	73 (58.4)	0.512
Prior transplantation	38 (23.03)	7 (17.5)	31 (24.8)	0.578
Deceased donor organ	101 (61.21)	25 (62.5)	76 (60.8)	0.848
Cold ischemia time (h)	11.00 (3.50, 31.12)	14.00 (4.20,24.00)	10.20 (3.50, 31.12)	0.215
HLA mismatches	3.09 ± 1.42	2.95 ± 1.47	3.14 ± 1.40	0.463
Peak PRA (%)	0.00 (0.00, 100.00)	0.00 (0.00, 70.00)	0.00 (0.00, 100.00)	**0.026**
Peak PRA ≥5%	35 (21.21)	3 (7.50)	32 (25.60)	**0.015**
Pre-transplant PRA (%)	0.00 (0.00, 98.00)	0.00 (0.00, 65.00)	0.00 (0.00, 98.00)	0.139
Pre-transplant PRA ≥5%	19 (11.52)	2 (5)	17 (13.60)	0.139
Etiology of ESRD				
Glomerulonephritis Interstitial nephritis Polycystic disease Htn/nephrosclerosis Diabetic nephropathy Other causes Unknowing etiology	30 (18.18) 21 (12.73) 10 (6.06) 11 (6.67) 12 (7.27) 35 (21.21) 46 (27.88)	6 (15) 6 (15) 2 (5) 4 (10) 1 (2.5) 11 (27.5) 10 (25)	24 (19.2) 15 (12) 8 (6.4) 7 (5.6) 11 (8.8) 24 (19.2) 36 (28.8)	0.639
Baseline Cr (mg/dL)^**^	1.63 ± 0.64	1.46 ± 0.56	1.69 ± 0.66	0.052
Delayed graft function	50 (30.3)	6 (15)	44 (35.2)	**0.021**

*Data are presented as mean ± SD, median (minimum, maximum), or n (%)*.

* *P-values indicated group differences for proteinuria <0.3 g/24 h compared with proteinuria ≥0.3 g/24 h*.

** *Three months after transplantation*.

*TG, transplant glomerulopathy; PU, proteinuria; Tx, transplantation; HLA, human leukocyte antigen; PRA, panel reactive antibody; ESRD, end-stage renal disease; Htn, hypertension; Cr, creatinine. Bold values indicated statistical significance*.

Fifty patients (30.3%) had experienced delayed graft function. Several baseline characteristics varied significantly between two groups categorized according to proteinuria (0.3 g/24 h) were therefore appropriately considered as confounders in multivariable analysis.

The first biopsy-proven TG was diagnosed at a median of 72 months (from 2 to 224 months) from transplantation to biopsy when the average age of kidney transplant recipients was 50.47 ± 14.82. The mean systolic blood pressure (SBP) and diastolic blood pressure (DBP) of TG patients were 140.01 ± 21.01 mmHg and 83.74 ± 15.38 mmHg, respectively, at the time of biopsy. Among the enrolled patients, 126 patients (126/165, 76.36%) showed antibody-mediated rejection (ABMR) (including 79 cases of chronic active ABMR and 47 cases of chronic ABMR), and 39 patients (39/165, 23.64%) were classified as isolated transplant glomerulopathy (iTG). The maintenance immunosuppressant regimens consisted mostly of tacrolimus (106/165, 64.24%) and mycophenolate mofetil (89/165, 53.94%). The majority of patients (121/165, 73.33%) were treated with angiotensin-converting enzyme inhibitor (ACEI) or angiotensin-receptor blocker (ARB) followed by calcium channel blocker (CCB) (78/165, 47.27%). TG patients with proteinuria had significantly higher SBP, DBP, and percentage of tacrolimus and CCB usage (*P* < 0.05) (Table 2).

**Table 2:** Patients' demographics and clinical characteristics at the time of biopsy for TG.

**Variables**	**Overall (*n =* 165)**	**PU <0.3 g/24 h (*n =* 40)**	**PU ≥0.3 g/24 h (*n =* 125)**	*P*-value^*^
Age at the time of Bx(years)	50.47 ± 14.82	53.05 ± 14.75	49.64 ± 14.80	0.206
Time from Tx to Bx (months)	72.00 (2.00, 224.00)	71.00 (8.00, 195.00)	72.00 (2.00, 224.00)	0.989
SBP (mmHg)	140.01 ± 21.01	131.78 ± 16.73	142.65 ± 21.61	**0.004**
DBP (mmHg)	83.74 ± 15.38	77.60 ± 12.13	85.70 ± 15.82	**0.001**
BMI	25.19 ± 4.58	25.34 ± 4.73	25.15 ± 4.55	0.817
PTDM	16 (9.70%)	1 (2.50%)	15 (12.00%)	**0.006**
Cr at the time of Bx (mg/dL)	2.67 ± 1.14	2.55 ± 1.30	2.70 ± 0.97	0.439
PU at the time of Bx (g/24 h)	1.38 ± 1.47	0.16 ± 0.70	1.77 ± 1.49	** <0.001**
Anti-HLA DSA^**^	124 (75.15)	32 (80)	92 (73.6)	0.415
ABMR diagnosis	126 (76.97)	33 (82.5)	93 (74.4)	0.294
**Immunosuppressant**				
CNIs (Tac/CyA)	106/48	20/16	86/32	**0.049**
MMF/MPA	89/72	23/16	66/56	0.594
Steroid-free regimen	32 (19.39)	8 (20.00)	24(19.20)	0.911
Anti-hypertension agents^***^				
ACEI/ARB	121 (73.33)	25 (62.5)	96 (76.8)	0.075
Beta-adrenergic blockers	65 (39.39)	13 (32.5)	52 (41.6)	0.305
Calcium channel blockers	78 (47.27)	11 (27.5)	67 (53.6)	**0.004**

*Data are presented as mean ± SD, median (minimum, maximum), or n (%)*.

* *P-values indicated group differences for proteinuria <0.3 g/24 h compared with proteinuria ≥ 0.3 g/24 h*.

** *Anti-HLA DSA included either anti-HLA class I or class II or both DSA*.

*** *More than 6 consecutive months treatment before biopsy*.

*Bx, biopsy; SBP, systolic blood pressure; DBP, diastolic blood pressure; BMI, body mass index; PTDM, post-transplant diabetes mellitus; HLA, human leukocyte antigen; DSA, donor-specific antibodies; ABMR, antibody-mediated rejection; CNI, calcineurin inhibitor, Tac, tacrolimus; CyA, cyclosporin A; MMF, mycophenolate mofetil; MPA, mycophenolic acid; ACEI, angiotensin-converting enzyme inhibitor; ARB, angiotensin-receptor blocker. Bold values indicated statistical significance*.

### Pathological Features

**Figure 2:**
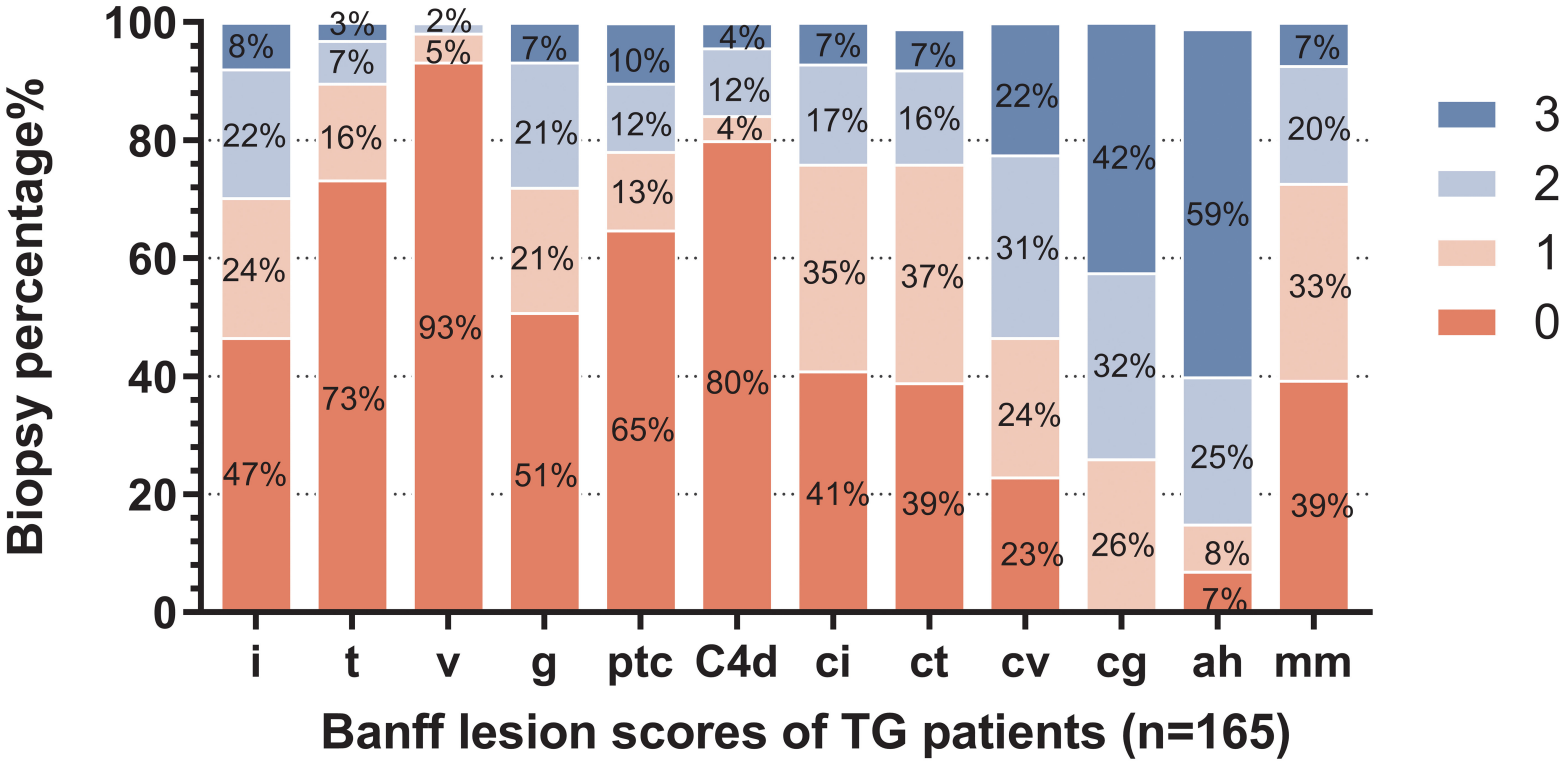
Distribution of Banff lesion scores for enrolled patients. The stacked bar graph denoted the Banff lesion scores distribution and the percentages of corresponding histological changes degrees of all enrolled biopsies (*n* = 165) in the study were shown. The abscissa scores represent I, interstitial inflammation; t, tubulitis; v, intimal arteritis; g, glomerulitis; ptc, peritubular capillaritis; C4d, endothelial cells C4d staining extent of peritubular capillary and medullary vasa recta; ci, interstitial fibrosis; ct, tubular atrophy; cv, vascular fibrous intimal thickening; cg, glomerular basement membrane double contours; ah, arteriolar hyalinosis; and mm, mesangial matrix expansion.

The distribution of histological changes in all TG patients is shown in Figure 2. Of enrolled 165 TG patients, 26.06% (*n* = 43) had a cg score of 1; 31.52% (*n* = 52) had a cg score of 2; 42.42% (*n* = 70) had a cg score of 3. More than half of TG patients had chronicity scores (ci, ct, cv, and cg) and i score as well as mm score. As features of microvascular inflammation (MVI), Banff lesion score g and ptc of TG patients were relatively low. The patients with positive staining for peritubular capillary C4d accounted for 20% (33/165).

When stratified by proteinuria, the Banff scores showed no statistically significant differences between patients with or without proteinuria (defined as ≥0.3 g/24 h) other than cg and mm scores (Table 3). cg score and mm score showed relatively higher trend in the group of TG patients with proteinuria, and there are also higher percentages of patients with lesion scores of more than 2 or 1, respectively.

**Table 3:** Histologic characteristics of enrolled biopsies.

**Variables**	**PU** ** <0.3 g/24 h (*****n=*** **40)**		**PU** **≥0.3 g/24 h (*****n=*** **125)**		*P*-value^*^
	**Score ≥1 n (%)**	**Mean ± SD**	**Score ≥1 n (%)**	**Mean ± SD**	
i score	23 (57.5)	1.03 ± 1.07	65 (52)	0.87 ± 0.98	0.401
t score	11 (27.5)	0.43 ± 0.81	33 (26.4)	0.39 ± 0.74	0.811
v score	4 (10)	0.10 ± 0.30	7 (5.6)	0.08 ± 0.35	0.746
g score	22 (55)	0.83 ± 0.90	59 (47.2)	0.84 ± 1.01	0.933
ptc score	11 (27.5)	0.60 ± 1.06	47 (37.6)	0.70 ± 1.03	0.612
C4d	4 (10)	0.25 ± 0.78	29 (23.2)	0.45 ± 0.88	0.178
ci score	22 (55)	0.90 ± 0.98	75 (60)	0.89 ± 0.90	0.943
ct score	25 (62.5)	1.00 ± 0.99	75 (60)	0.89 ± 0.90	0.504
cv score	30 (75)	1.45 ± 1.11	97 (77.6)	1.55 ± 1.07	0.604
cg score^**^	25 (62.5)	1.95 ± 0.85	97 (77.6)	2.23 ± 0.79	0.056
ah score	37 (92.5)	2.48 ± 0.93	117 (93.6)	2.34 ± 0.89	0.424
mm score	21 (52.5)	0.70 ± 0.76	79 (63.2)	1.03 ± 0.98	0.052

* *P-values indicated group differences by Student's t-test for proteinuria <0.3 g/24 h compared with proteinuria ≥0.3 g/24 h*.

** *Score ≥ 2 n (%)*.

*i score, interstitial inflammation; t score, tubulitis; v score, intimal arteritis; g score, glomerulitis; ptc score, peritubular capillaritis; C4d, endothelial cells C4d staining extent of peritubular capillary and medullary vasa recta; ci score, interstitial fibrosis; ct score, tubular atrophy; cv score, vascular fibrous intimal thickening; cg, score, glomerular basement membrane double contours; ah score, arteriolar hyalinosis; mm score, mesangial matrix expansion*.

**Figure 3:**
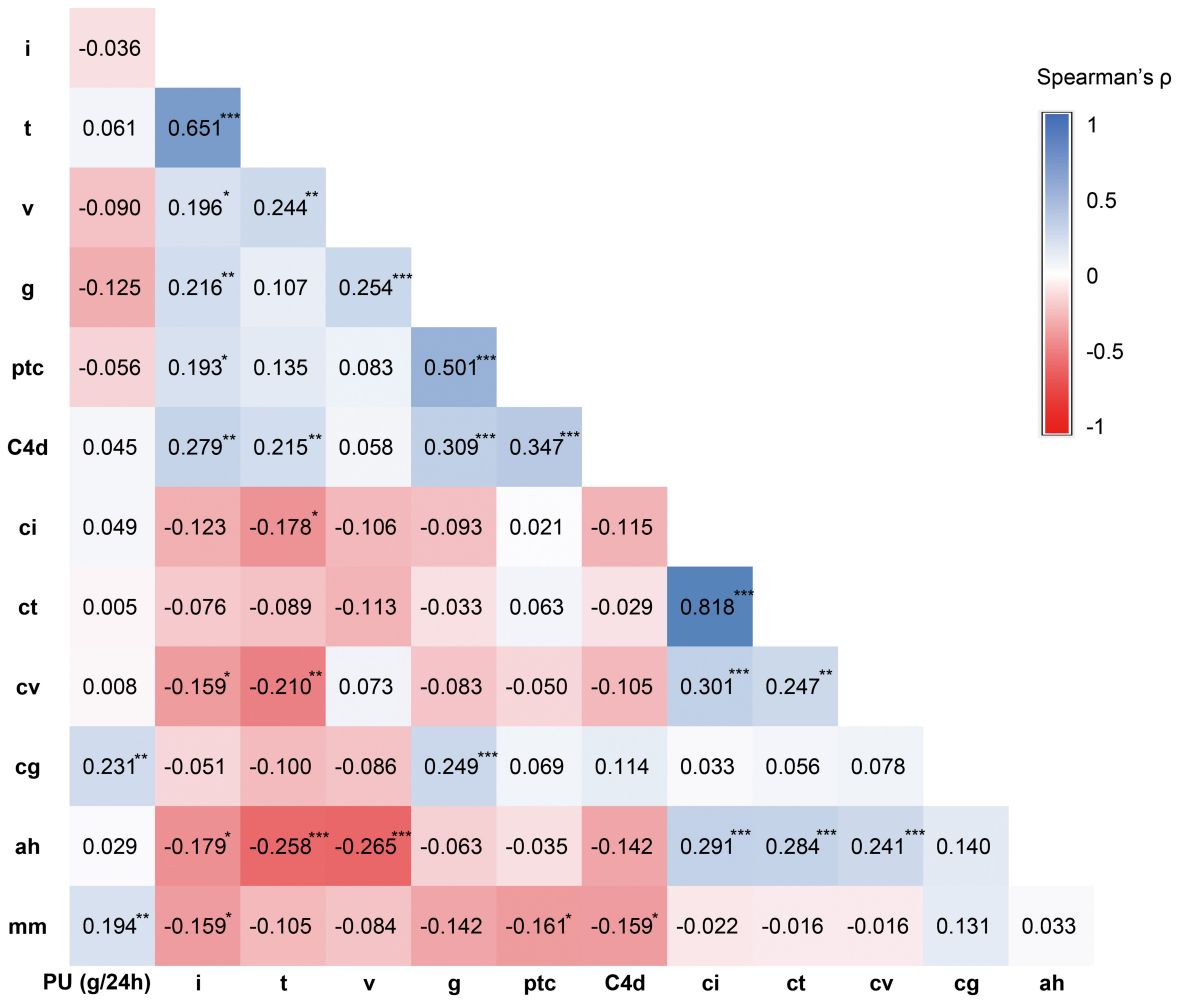
Correlation analysis results between proteinuria level and Banff lesion scores. The relationship between proteinuria level and Banff lesion scores were analyzed using Spearman correlation analysis. The correlation coefficient Spearman's ρ was shown in the graph. *P < 0.05, **P < 0.01, ***P < 0.001.

Banff i scores moderately correlated with Banff t (Spearman's ρ = 0.651, *P* < 0.001). As forms of MVI, Banff lesion score g and ptc correlated with each other significantly (ρ = 0.501, *P* < 0.001). Interstitial fibrosis/tubular atrophy scores ci and ct were highly correlated (ρ = 0.818, *P* < 0.001) (Figure 3).

### Histologic Lesions Associated With Proteinuria

**Figure 4:**
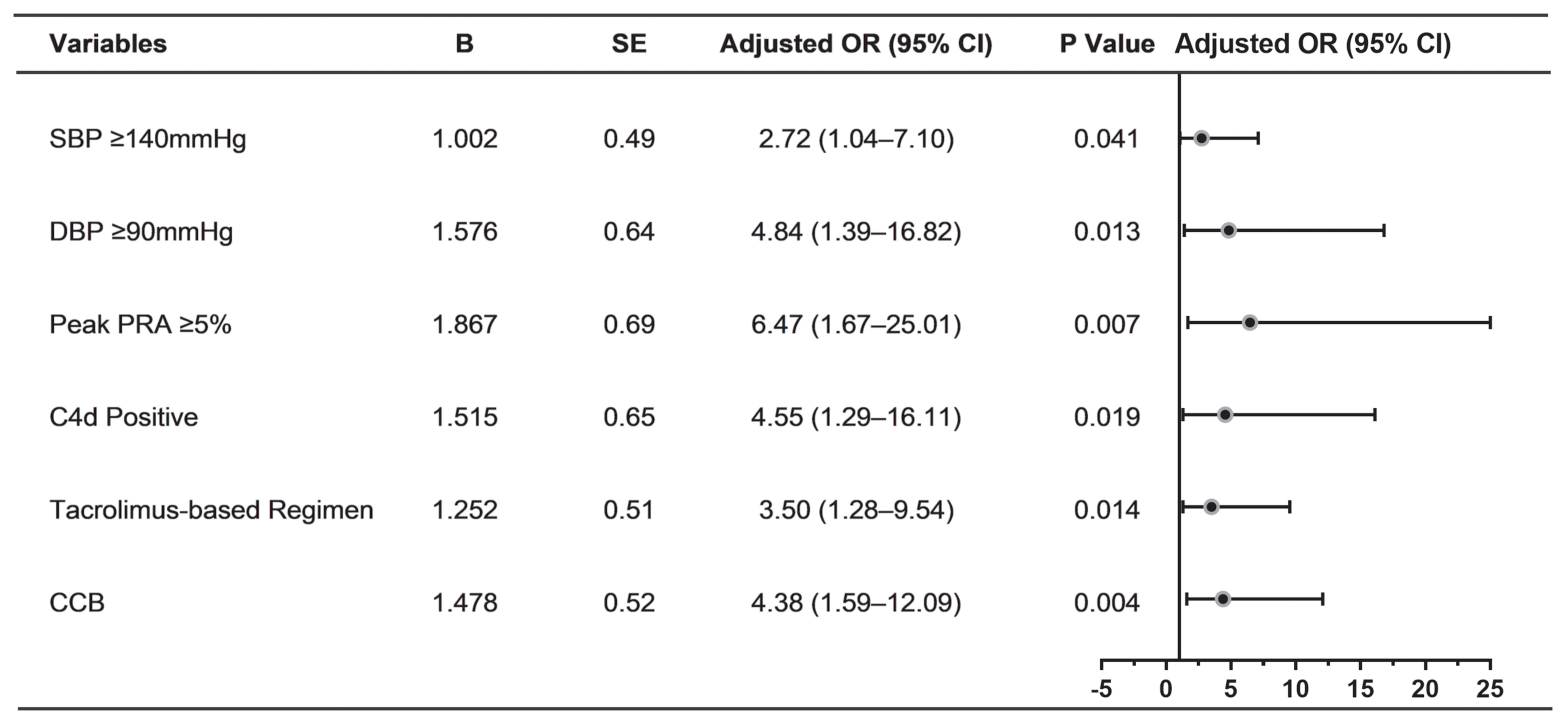
Determinants of proteinuria in multivariable logistic regression analysis (*n* = 165). Forest plot displaying odds ratios for proteinuria after multivariable logistic regression analysis. The following variables were included in the multivariable model: Peak PRA ≥ 5%, delayed graft function, SBP ≥ 140 mmHg, DBP ≥ 90 mmHg, CNI regimens, use of ACEI/ARB, use of CCB, positive C4d staining, cg score ≥2. SE, standard error; OR, odds ratio; CI, confidence interval; SBP, systolic blood pressure; DBP, diastolic blood pressure; PRA, panel reactive antibody; CCB, calcium channel blocker; CNI, calcineurin inhibitor; ACEI, angiotensin-converting enzyme inhibitor; ARB, angiotensin-receptor blocker.

The level of proteinuria best correlated with Banff lesion score cg (Spearman's ρ = 0.231, *P* = 0.003) and mm (ρ = 0.194, *P* = 0.012), whereas the interstitial fibrosis/tubular atrophy scores, C4d scores (ρ = 0.045, *P* = 0.1), and cv (ρ = 0.008, *P* = 0.915) were unrelated (Figure 3). None of Banff lesion scores showed positive result in univariable regression analysis of proteinuria (Table 4). Positive C4d staining was found to be a significant pathologic risk factor for proteinuria as determined by multivariable regression analysis (Figure 4).

**Table 4:** Univariable logistic regression analysis for the predictors of proteinuria.

**Variables**	**Category**	**PU incidence (%)**	**Odds ratio (95%CI)**	***P*-value**
**Demographics and clinical characteristics**				
Gender	Male/Female	77.66/73.24	1.27 (0.62–2.60)	0.512
Age at Tx (years)	≥60/18–59	83.87/73.88	1.84 (0.66–5.16)	0.247
Previous kidney Tx	y/*n*	81.58/74.01	1.67 (0.74–3.73)	0.215
Donor types	Non–/living donor	75.25/76.56	0.93 (0.45–1.94)	0.848
Cold ischemia time (h)	≥12/ <12 h	65.22/83.64	0.37 (0.14–0.94)	**0.036**
HLA mismatches	≥3/0–2	78.09/71.70	1.46 (0.71–3.03)	0.308
Peak PRA ≥5%	y/*n*	91.43/71.54	4.42 (1.28–15.32)	**0.019**
Pre–Tx PRA ≥5%	y/*n*	89.47/73.97	2.99 (0.66–13.55)	0.155
Delayed graft function	y/*n*	88/71.3	2.95 (1.14–7.63)	**0.025**
Baseline Cr (mg/dL)	≥1.5/1–1.5	80.68/68.12	1.01 (0.50–2.07)	0.974
Time from Tx to Bx (m)	≥60/0–60	77/73.85	1.19 (0.58–2.44)	0.644
SBP (mmHg)	≥140/90–140	85.54/63.52	3.07 (1.43–6.58)	**0.004**
DBP (mmHg)	≥90/50–90	92.16/68.42	5.36 (1.80–15.93)	**0.003**
PTDM	y/*n*	12.00/2.5	5.32 (0.68–41.60)	0.111
Calcineurin inhibitors	Tac/CyA	81.13/66.67	2.15 (0.99–4.66)	0.052
Antiproliferative agents	MMF/MPA	74.16/77.78	1.18 (0.61–2.29)	0.617
Steroid–free regimen	y/*n*	75/75.94	1.05 (0.43–2.57)	0.911
BMI	≥25/	75/77.66	0.86 (0.42–1.80)	0.694
Cr at the time of Bx (mg/dL)	≥1.5/1–1.5	76.82/64.29	1.84 (0.58–5.85)	0.301
Age at Bx (years)	≥60/18–59	83.02/72.32	1.87 (0.82–4.28)	0.138
Anti–HLA DSA	y/*n*	74.19/80.49	0.70 (0.29–1.67)	0.417
Anti–hypertension agents^***^				
ACEI/ARB	y/*n*	79.34/65.91	1.99 (0.93–4.26)	0.078
Beta–adrenergic blockers	y/*n*	79.69/73.27	1.48 (0.71–3.09)	0.302
Calcium channel blockers	y/*n*	85.71/67.05	2.95 (1.36–6.37)	**0.006**
Etiology	ABMR/iTG	73.81/82.05	0.67 (0.28–1.59)	0.358
**Banff lesion scores**				
i score	≥1/0	73.86/77.92	0.80 (0.39–1.64)	0.544
t score	≥1/0	75/76.03	0.95 (0.43–2.10)	0.891
v score	≥1/0	63.64/76.62	0.53 (0.15–1.93)	0.338
g score	≥1/0	72.84/78.57	0.73 (0.36–1.50)	0.391
ptc score	≥1/0	81.03/72.90	1.59 (0.73–3.48)	0.246
C4d	≥1/0	87.88/72.73	2.72 (0.89–8.28)	0.078
ci score	≥1/0	77.32/73.53	1.23 (0.60–2.52)	0.576
ct score	≥1/0	75/76.92	0.90 (0.43–1.87)	0.778
cv score	≥1/0	76.38/73.68	1.16 (0.50–2.65)	0.734
cg score	≥2/1	79.51/65.12	2.08 (0.97–4.47)	0.061
ah score	≥1/0	75.97/72.73	1.19 (0.30–4.70)	0.808
mm score	≥1/0	79/70.77	1.55 (0.76–3.19)	0.230

*Univariable logistic regression analysis was used to select possible predictors of proteinuria (PU ≥0.3 g/24 h). Incidence of PU was shown in different categories TG patients. P-values indicated the significance associated with proteinuria. CI, confidence interval; iTG, isolated transplant glomerulopathy. Bold values indicated statistical significance*.

*** *More than 6 consecutive months treatment before biopsy*.

### Risk Factors of Proteinuria in TG Patients

The prevalence of proteinuria for enrolled TG patients was 75.76% (in male 77.66% and female 73.24%, respectively, Supplementary Table 1). Using univariable logistic regression, cold ischemia time ≥12 h, Peak PRA ≥5%, delayed graft function, SBP ≥140 mmHg, DBP ≥90 mmHg, use of CCB were significantly associated with a greater likelihood of proteinuria (Table 4).

When significant variables in univariable analysis and those potential risk factors (*P* < 0.1) were analyzed by multivariable logistic regression, peak PRA ≥5%, SBP ≥ 140 mmHg, DBP ≥90 mmHg, tacrolimus-based regimen, use of CCB, positive C4d staining were independently associated with the development of proteinuria in TG patients (Figure 4).

**Figure 5:**
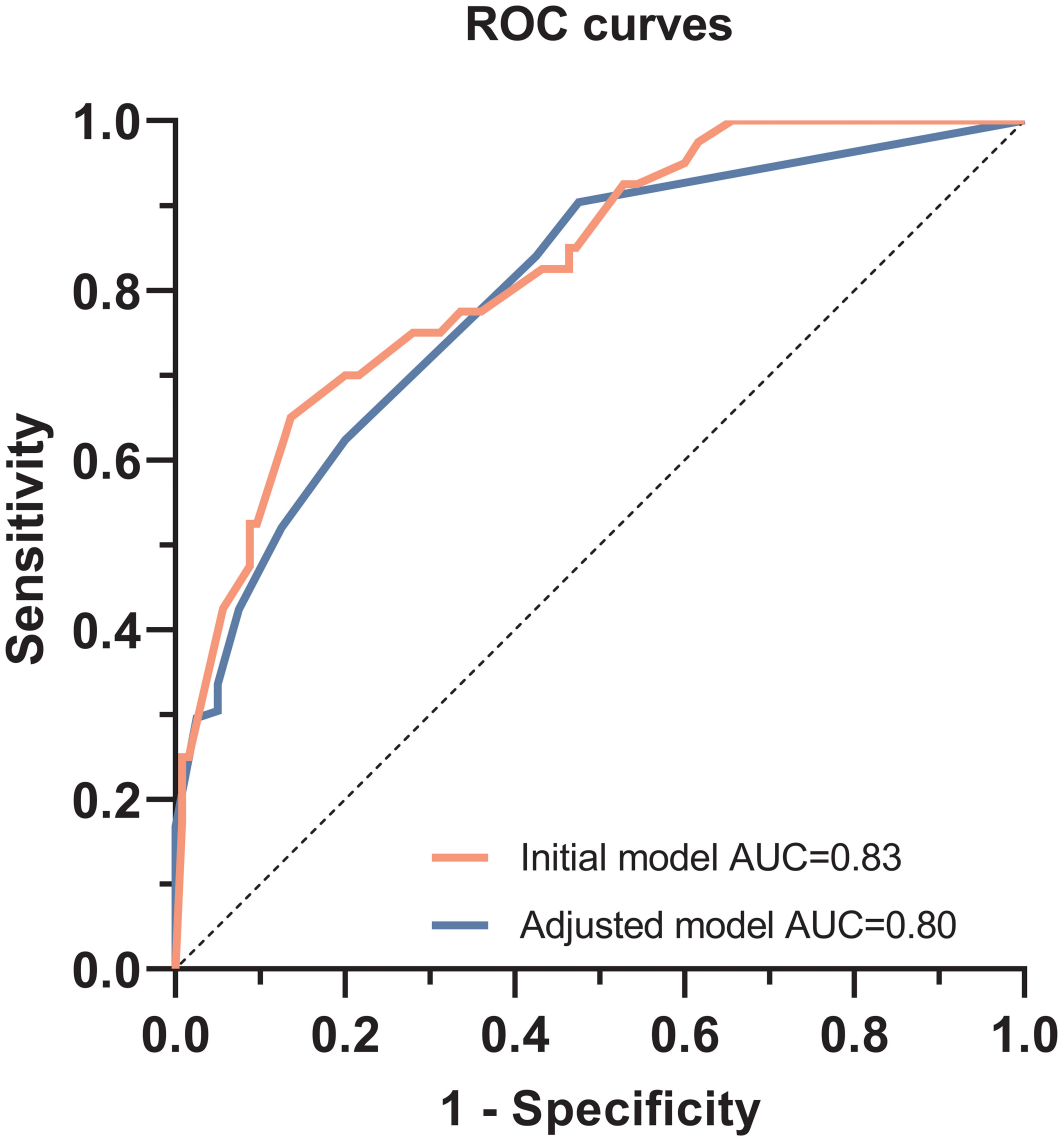
Receiver operating characteristic curves to evaluate the predictive value of the multivariable logistic regression model for predicting proteinuria in TG patients. For the initial multivariable logistic regression model, the area under the curve (AUC) was 0.83 (95% CI, 0.76–0.90, *P* < 0.001). After bootstrap replication, AUC was 0.80 (95% CI, 0.72–0.87, *P* < 0.001).

**Figure 6:**
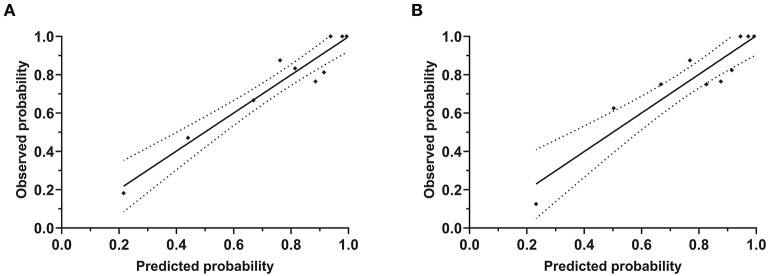
Calibration plot of risk estimation for proteinuria prediction model. These plots showed the calibration accuracy of the models between observed and predicted probabilities (Hosmer Lemeshow test). The X-axis and Y-axis indicated the predicted and observed probabilities of proteinuria from the initial model **(A)** and adjusted model **(B)**, respectively.

The patient's risk score was generated using coefficients obtained from the multivariable logistic regression analysis as follow: risk score = (1.002×SBP ≥140 mmHg) + (1.576×DBP ≥90 mmHg) + (1.867×peak PRA ≥5%) + (1.515×C4d = positive) + (1.252×tacrolimus-based regime*n* = yes)) + (1.478×use of CCB = yes)-1.56 (23). This model yielded a good fit to the data (Hosmer Lemeshow test, χ^2^= 7.38, df = 8, *P* = 0.497) and excellent discrimination (AUC = 0.83, 95% CI 0.76–0.90). The internal validation using bootstrap with 2,000 repetitions showed a similar AUC of 0.80 (95% CI 0.72–0.87) (Figure 5). This model also showed favorable consistency between the predicted proteinuria and the actual probability of observation with a slope of 0.995 and an intercept of 0.004 in the calibration line. The calibration performance remained similar after adjusted for optimism in an internal validation (slope = 1.007; intercept = −0.004) (Figure 6).

## Discussion

In this study, we investigated the clinical and histological features of TG patients and further identified the risk factors of proteinuria in TG patients based on almost two decades of consecutive patients' data. Based on the model developed using multivariable logistic regression, we found SBP ≥140 mmHg, DBP ≥90 mmHg, peak PRA ≥5%, positive C4d staining, tacrolimus-based regimen, and CCB usage were independent risk factors for proteinuria. After internal validation, this model still afforded high discrimination (0.80, 95% CI 0.72–0.87) and good calibration (slope of 1.007 and intercept of −0.004).

Proteinuria is very common after kidney transplantation, and it is also one of the most important manifestations of TG. The incidence of proteinuria (defined as urine protein output ≥0.3 g/24 h) in our study was 75.76%. Due to the relatively low threshold for defining proteinuria, the incidence of proteinuria was relatively high compared to a previous study, in which proteinuria was present in 40% out of 55 TG patients with cut-off defined as 0.5 g/day (4). Since most of TG patients were diagnosed more than 1 year after kidney transplantation, and patients diagnosed with TG within 1 year after transplantation (*n* = 10) had undergone at least 9 months of dialysis treatment in our study, proteinuria was not considered to originate from the native kidneys (24). Proteinuria is the marker of glomerular injury, while the main pathologic feature of TG is endothelial injury with GBM double contours. TG can cause proteinuria, similar to other proteinuric factors, it eventually leads to allograft loss (3, 4).

Consistent with previous studies, the severity of proteinuria was significantly correlated with Banff lesion score cg and mm though both of them were not significant in univariable and multivariable logistic regression analysis (13, 25, 26). This might due to the lower cut-off value for the diagnosis of proteinuria in this study. From a pathological point of view, as the extent of GBM multilamination increases, its role as a barrier against protein leakage decreases, which in turn leads to proteinuria (2). Mesangial matrix expansion usually accompanies the TG glomerular changes, gradually increases over the development of TG, but much later than endothelial and subendothelial abnormalities (26, 27). This might be the reason for its association with proteinuria.

Posttransplant hypertension, defined as a blood pressure >140/90 mmHg, was observed in 57.58% enrolled TG patients in the present study. Odds of proteinuria were up to quadruple as high in those with hypertension compared to those with normal blood pressure. Hypertension in kidney transplant recipients has been shown to be associated with proteinuria (28, 29). This association may be due to the pathological changes that lead to proteinuria, like TG or interstitial fibrosis/tubular atrophy which can also cause hypertension. While, high blood pressure can damage the kidney *per se*, and patients with hypertension can also develop proteinuria without evidence of intrinsic renal disease (30). For hypertensive TG patients, in addition to the proteinuria that may be caused by TG, it is appealing to conceive that high filtration caused by hypertension might further aggravate proteinuria. Furthermore, proteinuria can in turn contribute to the development of hypertension in kidney transplant recipients (31). The above facts and inferences give us more reason to strengthen blood pressure management for patients with TG, especially those with proteinuria.

Similar to other kidney transplant patients, the application of CCB therapy increased the risk of proteinuria in TG patients. This may be due to the vasodilatory effect of dihydropyridine CCB that increases intraglomerular pressure and thus leads to proteinuria (31, 32). Yet both CCB usage and proteinuria information were obtained at the same time, the issue of simultaneity should also be considered. In the present study, the application of ACEI/ARB had no significant effect on the production of proteinuria. Conversely, the proportion of ACEI/ARB application was relatively high in proteinuric TG patients (76.8 vs. 62.5%) although no statistical difference was reached (*P* = 0.075). It is plausible that the use of ACEI/ARB, to a certain extent, represents the potential existence of proteinuria or cardiovascular diseases which may also associate with proteinuria.

Contrary to the results of previous studies, C4d deposition in PTC was identified as a risk factor for proteinuria in TG patients in our study (13, 25). Peritubular capillary C4d usually represents active antibody-mediated rejection, all C4d positive patients showed evidence of DSA before the biopsy in our study (33). Compared with 72.73% of proteinuric proportion in TG patients without C4d deposition, 87.88% C4d positive TG patients had proteinuria. Based on these facts, it is reasonable to identify a link between C4d deposition and the development of proteinuria. The discrepancy between the present and previous studies could possibly due to the differences in patient enrollment criteria and definitions of proteinuria.

Up to 91.43% of TG patients with peak PRA ≥5% had proteinuria, previous reports also noted the association of PRA with proteinuria (28, 34). Interestingly, the relationship with proteinuria was found only in TG patients with high peak PRA rather than pre-transplant PRA in our study. Although the detailed mechanism needs to be further verified, special attention should be paid to these patients.

The impact of immunosuppressants on proteinuria requires specific analysis. Unlike CNIs and mycophenolate compounds, mTOR inhibitors including sirolimus and everolimus are clearly related to proteinuria (10, 35). Thus, these patients were excluded from our analysis. A higher proportion of tacrolimus-based regimen in proteinuric TG patients was found in our study, and further multiple logistic regression indicated that tacrolimus was a risk factor for proteinuria in TG patients. For kidney transplant recipients with recurrent MPGN, tacrolimus did not control proteinuria compared to cyclosporin, despite the similar changes in allograft hemodynamics (36). However, Halimi et al. found that cyclosporine was significantly associated with urinary non-albumin proteins (28). Due to the lack of blood concentration information, it is difficult to determine whether the different effect of these two types of CNIs on proteinuria is due to the different regimens or CNI toxicity. Meanwhile, the possibility of reverse causality cannot be excluded. Further research is needed to confirm those.

The present study has several limitations. First, although our study involves one of the largest biopsy-proven TG patients to date, the sample size is still relatively small. Second, due to the lack of external validation strength of our model is unknown for another independent patient population, despite bootstrap is considered the most reliable technique to validate internally for the logistic regression model (37). Third, different treatment modalities for TG, though with limited effect on graft survival, associations between therapeutic interventions and proteinuria should also investigate.

Another disadvantage is the fact that no electron microscopy data were available. Regardless of anything else, this must be mentioned that all the data were acquired either at the of transplantation or at the time of biopsy it would be hard to infer the association between “risk factors” and proteinuria. Therefore, further studies should be performed to determine the causation.

Taken together, our findings demonstrate that hypertension, peak PRA ≥5%, positive C4d staining, tacrolimus-based regimen, and CCB usage were significantly associated with proteinuria in TG patients. Our multivariable logistic regression model showed good predictive accuracy and optimal calibration, it may assist clinicians in deciding whether a further adjustment should be taken after further prospective study confirmation.

## Data Availability Statement

The raw data supporting the conclusions of this article will be made available by the authors, without undue reservation.

## Ethics Statement

Ethical review and approval was not required for the study on human participants in accordance with the local legislation and institutional requirements. Written informed consent for participation was not required for this study in accordance with the national legislation and the institutional requirements.

## Author Contributions

QZ, KB, BR, and KW participated in research design. QZ, KB, FH, MD, MN, MM, WD, BR, and KW participated in the writing of the paper. QZ, KB, DS, FK, BR, and KW participated in the performance of the study. QZ, KB, and KW participated in data analysis. All authors contributed to the article and approved the submitted version.

## Conflict of Interest

The authors declare that the research was conducted in the absence of any commercial or financial relationships that could be construed as a potential conflict of interest.
