# Supplementary material for: Clinicopathologic Features and Risk Factors of Proteinuria in Transplant Glomerulopathy
Source: Front Med (Lausanne). 2021 Jul 2;8:666319. doi: 10.3389/fmed.2021.666319 (PMC8283120; doi:10.3389/fmed.2021.666319)
Supplement: Supplementary file 1 [file Table_1.docx]

**Table S1 Level of proteinuria at the time of biopsy for transplant glomerulopathy**

| **Urine protein**  **(g/24 hour)** | **Overall**  **(n=165)** | **Male**  **(n=94)** | **Female**  **(n=71)** | **P value** |
| --- | --- | --- | --- | --- |
| Mean (range) | 1.38±1.47  (0.05-6.9) | 1.50±1.51  (0.05-6.90) | 1.21±1.41  (0.05-6.84) | 0.206 |
| 0-0.3 | 40 (24.24%) | 21 (22.34%) | 19 (26.76%) | 0.175 |
| 0.3-1 | 52 (31.52%) | 25 (26.60%) | 27 (38.03%) |  |
| 1-3 | 54 (32.73%) | 34 (36.17%) | 20 (28.17%) |  |
| >3 | 19 (11.52%) | 14 (14.89%) | 5 (7.04%) |  |
